# Highly Efficient Lightweight Flexible Cu(In,Ga)Se_2_ Solar Cells with a Narrow Bandgap Fabricated on Polyimide Substrates: Impact of Ag Alloying, Cs and Na Doping, and Front Shallow Ga Grading on Cell Performance

**DOI:** 10.1002/smsc.202400404

**Published:** 2024-12-16

**Authors:** Yukiko Kamikawa, Jiro Nishinaga, Takeshi Nishida, Shogo Ishizuka

**Affiliations:** ^1^ Global Zero Emission Research Center National Institute of Advanced Industrial Science and Technology (AIST) Tsukuba Ibaraki 305‐8568 Japan

**Keywords:** bottom cells, chalcopyrite, solar cell capacitance simulator, surface fields, thin‐film solar cells

## Abstract

Herein, lightweight, flexible Cu(In,Ga)Se_2_ (CIGS) solar cells with a narrow bandgap of ≈1 eV are grown on polyimide substrates. The poor performance of the CIGS solar cells owing to a low growth temperature (≈400 °C) is considerably improved via Ag alloying, Na doping using alkali‐silicate‐glass thin layers (ASTLs) and the CsF postdeposition treatment (CsF‐PDT), and front shallow Ga grading (surface field; SF). Along with improved device process, a notably high conversion efficiency of 21.2%, low *V*_OC_ deficit of 0.346 V, and high *J*_SC_ of ≈40 mA cm^−2^ are achieved. Ag alloying and Na doping using ASTLs predominantly improve the CIGS bulk quality, while the CsF‐PDT and SF reduce carrier recombination at the CIGS/CdS interface and vicinity. Device simulations reveal that the SF increases the electrical field at the CIGS surface under the forward bias voltage close to *V*_OC_ owing to electron injection from the CdS side, which increases the chemical potential. Thus, the SF effectively repulses holes and improves the interfacial property. Device simulations also reveal that a high CIGS absorber's quality is prerequisite to benefit from the SF. Thus, CIGS solar cells showing improved bulk quality due to optimum alkali doping and Ag alloying considerably benefit from the SF.

## Graphical abstract

Lightweight, flexible Cu(In,Ga)Se_2_ (CIGS) solar cell with high efficiency of 21.2% and narrow bandgap of ≈1.03 eV is fabricated on polyimide substrate. Ag and alkali metals’ doping (Na, Cs) improve bulk and interfacial quality of CIGS solar cells. Shallow front Ga grading further reduces carrier recombination at the CIGS/CdS by increasing electrical field repulsing holes.

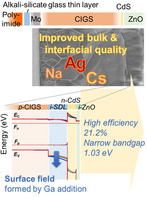

## 1 Introduction

Photovoltaics (PVs) are a promising solution to reduce greenhouse gas (GHG) emissions and meet global energy needs; hence, the demand for PVs is increasing. PVs have been recognized as a “no regret option” for the next decade owing to its easy availability, cost‐effectiveness, and alignment with long‐term sustainable development goals.^[^ ^1^ ^]^ Accordingly, the demand for lightweight solar cells with flexible characteristics has also increased.^[^ ^2^, ^3^ ^]^ Highly efficient tandem solar cells can reduce the levelized cost of PV energy and accelerate the global installation of PV systems.^[^ ^4^ ^]^ However, building‐integrated PVs and applications such as vehicle‐integrated PVs, stratospheric airships, and space applications require high specific power (kW kg^−1^)^[^ ^5^, ^6^, ^7^, ^8^, ^9^ ^]^ and flexibility to fit curved surfaces. Cu(In, Ga)Se_2_ (CIGS)‐based thin‐film solar cells are potential candidates for lightweight and flexible solar cells. When used as single solar cells, the efficiency of CIGS solar cells surpasses 23% and 22% on rigid and flexible substrates, respectively.^[^ ^10^, ^11^, ^12^, ^13^ ^]^ CuInSe_2_ (CIS) and low‐Ga‐content CIGS solar cells with narrow energy bandgaps of ≈1.0 eV are potential candidates for bottom cells used in tandem solar cells.^[^ ^14^, ^15^, ^16^, ^17^, ^18^ ^]^ The lightweight nature, flexibility,^[^ ^19^, ^20^ ^]^ and high radiation tolerance^[^ ^21^, ^22^, ^23^ ^]^ of CIGS bottom cells will be greatly advantageous. When the top cells of tandem solar cells have the same features, the advantages will be those of the tandem solar cells.^[^ ^14^, ^15^, ^16^, ^17^, ^18^, ^19^, ^20^, ^21^, ^22^, ^23^ ^]^ Several materials such as polyimide (PI), metals, and ceramics are used as substrates in flexible CIGS solar cells.^[^ ^3^, ^19^, ^20^ ^]^

Herein, PI films were formed on supporting glass substrates by spin coating of varnish followed by curing.^[^ ^24^ ^]^ CIGS solar cells were fabricated on the PI films via a low‐temperature processes of ≤400 °C.^[^ ^25^ ^]^ Generally, CIGS solar cells with an absorber fabricated via a low‐temperature process exhibit poor performance, which can be addressed using various approaches such as the introduction of light and heavy alkali metals,^[^ ^24^, ^26^, ^27^, ^28^, ^29^, ^30^, ^31^ ^]^ Ag alloying,^[^ ^10^, ^32^, ^33^, ^34^, ^35^, ^36^ ^]^ and engineering of conduction‐ and/or valence‐band (*E*_c_, *E*_v_) gradings.^[^ ^37^, ^38^, ^39^ ^]^ Among these, Ag alloying and CsF postdeposition treatment (CsF‐PDT) were employed herein for fabricating CIGS absorbers. Moreover, an alkali‐silicate‐glass thin layer (ASTL) was introduced as a Na source underneath back contact (Mo). Furthermore, a front shallow Ga grading (hereinafter referred to as the surface field [SF]) was introduced in the CIGS absorbers. The effects of Ag alloying, CsF‐PDT, ASTL, and SF were elucidated via experiments and device simulations.

## 2 Results and Discussion

### 2.1 CIGS Absorber Layer and CIGS Solar Cell's Characteristics

#### 2.1.1 Influence of Ag Alloying, the CsF‐PDT, and the ASTL on Crystal Morphology and the Ga Grading

**Figure 1:**
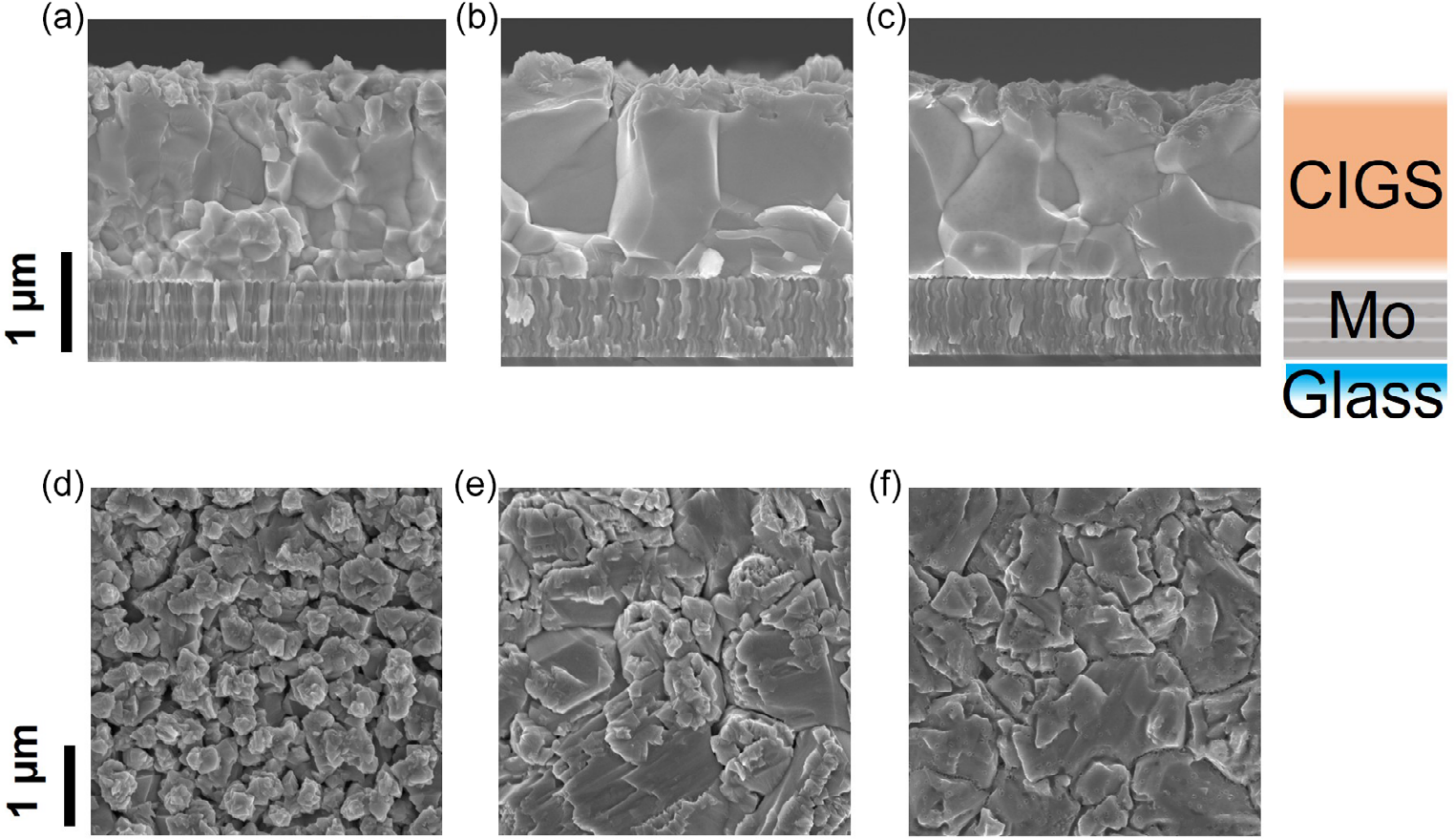
Cross‐sectional SEM images for the CIGS absorber layer fabricated on Mo‐coated SLG substrate a) without Ag alloying or CsF‐PDT, b) with Ag alloying and without CsF‐PDT, c) with Ag alloying and CsF‐PDT, and d–f) plan‐view images.

First, the influence of Ag alloying, CsF‐PDT, and ASTL on the crystal morphology and Ga grading was evaluated. For this purpose, an SF was introduced in all samples discussed in Section 2.1.1 and 2.1.2. It is because impacts of Ag alloying, CsF‐PDT, and ASTL on device performances are more pronounced with the SF (e.g., Section 2.1.3). The impact of the SF is separately discussed in Section 2.1.3 and 2.2.1. **Figure** **1** shows the cross‐sectional scanning electron microscopy (SEM) images of the CIGS absorber layer deposited on the reference Mo‐coated soda–lime glass (SLG) substrate at ≈400 °C (a) without Ag alloying or CsF‐PDT, (b) with Ag alloying and without CsF‐PDT, (c) with Ag alloying and CsF‐PDT, and (d)–(f) plan‐view images. The samples were rinsed with water before SEM measurements. Figure S1, Supporting Information, shows that the samples subjected to CsF‐PDT exhibited precipitation‐like patterns before rinsing, which were removed after rinsing with water. The grain sizes in the CIGS absorber layer without Ag alloying were very small within several hundred nanometer range (Figure 1a,d) owing to low‐temperature growth. With the addition of Ag precursor, the grain sizes enlarged to the order of micrometers.^[^ ^13^, ^32^, ^36^ ^]^ The grain sizes did not change after the absorber layer underwent CsF‐PDT.

**Figure 2:**
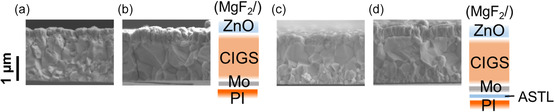
Cross‐sectional SEM images of the CIGS layer fabricated a) without Ag alloying or CsF‐PDT, b) with Ag alloying and CsF‐PDT on Mo‐coated PI substrates, c) with Ag alloying and CsF‐PDT on Mo‐coated and 50‐ nm thick ASTL‐coated PI substrates, and d) with Ag alloying and CsF‐PDT on Mo‐coated and 100 nm thick ASTL‐coated PI substrates.

**Figure** **2** shows the cross‐sectional SEM images of the CIGS absorber layer fabricated (a) without Ag alloying or CsF‐PDT, (b) with Ag alloying and CsF‐PDT on Mo‐coated PI substrates, (c) with Ag alloying and CsF‐PDT on Mo‐coated and 50 nm thick ASTL‐coated PI substrates, and (d) with Ag alloying and CsF‐PDT on Mo‐coated and 100 nm thick ASTL‐coated PI substrates. Similar to SLG substrates, grain growth in the CIGS absorber layer deposited on PI substrates enhanced by introducing the Ag precursor. Moreover, upon ASTL deposition,^[^ ^24^, ^40^ ^]^ the grain size of the bottom part decreased^[^ ^40^ ^]^ probably due to inhibited grain growth by introduction of Na through the ASTL.^[^ ^30^, ^40^, ^41^ ^]^

**Figure 3:**
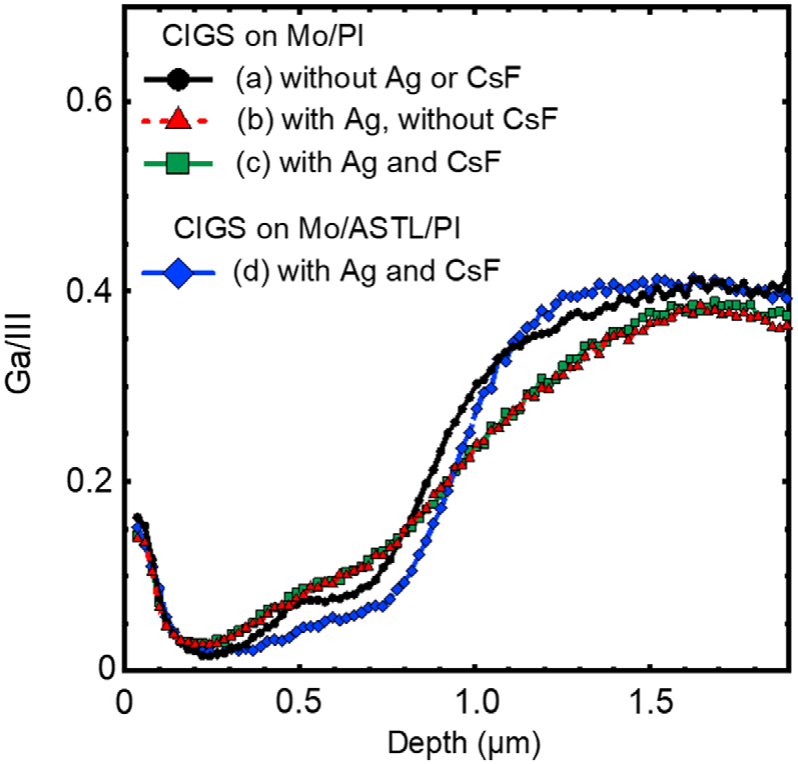
Ga/III in the CIGS absorber layer fabricated (a) without Ag alloying or CsF‐PDT, (b) with Ag alloying and without CsF‐PDT, (c) with Ag alloying and CsF‐PDT on the Mo‐coated PI substrate, and (d) with Ag alloying and CsF‐PDT on the Mo‐coated and 50 nm thick ASTL‐coated PI substrates evaluated via SIMS.

**Figure** **3** shows the Ga/(In + Ga) ratio, i.e., (Ga/III), in the CIGS absorber layer fabricated (a) without Ag alloying or CsF‐PDT, (b) with Ag alloying and without CsF‐PDT, (c) with Ag alloying and CsF‐PDT on the Mo‐coated PI substrate, and (d) with Ag alloying and CsF‐PDT on the Mo‐coated and 50 nm thick ASTL‐coated PI substrates evaluated via secondary‐ion mass spectrometry (SIMS). The introduction of Ag enhanced the interdiffusion of Ga and In and moderated the Ga/III slope.^[^ ^36^ ^]^ The Ga/III depth profile was not impacted by CsF‐PDT. The Ga/III slope steepened with introduction of the ASTL due to hindered Ga and In interdiffusion.^[^ ^30^, ^40^, ^41^, ^42^ ^]^ The Ga/III depth profiles of CIGS absorber layers deposited on the Mo and 100 nm thick ASTL‐coated PI substrates were almost identical with that of the layer deposited on 50 nm thick ASTL (Figure S2, Supporting Information).

#### 2.1.2 Influence of Ag Alloying, the CsF‐PDT, and the ASTL on Device Performance

**Figure 4:**
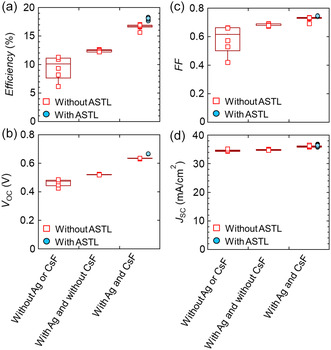
a) Conversion efficiency, b) *V*_OC_, c) FF, d) *J*_SC_ of CIGS solar cells fabricated under three growth conditions: without Ag alloying or CsF‐PDT, with Ag alloying but without CsF‐PDT, and with Ag alloying and CsF‐PDT. The data for the solar cells without the ASTL and with 50 nm thick ASTL are marked as squares and circles, respectively. No AR coating was applied on the devices.

**Figure 5:**
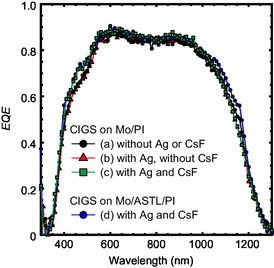
EQE spectra of CIGS solar cells fabricated (a) without Ag alloying or CsF‐PDT, (b) with Ag alloying and without CsF‐PDT, (c) with Ag alloying and CsF‐PDT on the Mo‐coated PI substrate, and (d) with Ag alloying and CsF‐PDT on the Mo‐coated and 50 nm ASTL‐coated PI substrates. The solar cells were without any AR coating.

Next, the effects of Ag alloying, the CsF‐PDT, and the ASTL on the device performance were evaluated. **Figure** **4** shows the (a) conversion efficiency, (b) open‐circuit voltage (*V*_OC_), (c) fill factor (FF), and (d) short‐circuit current density (*J*_SC_) of the CIGS solar cells fabricated under three growth conditions: without Ag alloying or CsF‐PDT, with Ag alloying but without CsF‐PDT, and with Ag alloying and CsF‐PDT. The data for the solar cells without the ASTL and with 50 nm thick ASTL are marked as squares and circles, respectively. An antireflective (AR) coating was not applied on these devices. By introduction of Ag alloying, ASTL deposition, and CsF‐PDT, the conversion efficiency of the CIGS solar cells improved, mainly due to improved *V*_OC_ and FF. Without Ag alloying or CsF‐PDT, *V*_OC_ was as low as 0.49 V. With Ag alloying, CsF‐PDT, and 50 nm thick ASTL deposition, *V*_OC_ increased to 0.67 V. These approaches did not considerably impact *J*_SC_ (Δ*J*_SC_ < 2 mA cm^−2^); however, a detailed investigation of the external quantum efficiency (EQE) (**Figure** **5**) revealed that the CsF‐PDT slightly increased the EQE in the short‐wavelength regions (400–550 nm). As the CdS deposition method was unchanged, this change might correspond to reduced carrier recombination at the CIGS/CdS interface and/or its vicinity. Although an obvious variation was observed in the Ga/III depth profiles in those samples (Figure 3), the Ga/III depth profile variation moderately impacted the EQE. Hara et al. reported that the majority of light was absorbed in the front region (≈0.8 μm), whereas negligible absorption was observed in the bottom region (0.8–2 μm). The bottom layer with a higher Ga content played a dominant role as a back‐surface field (BSF) with conduction‐band grading.^[^ ^43^ ^]^ As shown in Figure 3, the Ga/III depth profile considerably varies in the bottom region (0.8–2 μm) that acts as the BSF.

**Figure 6:**
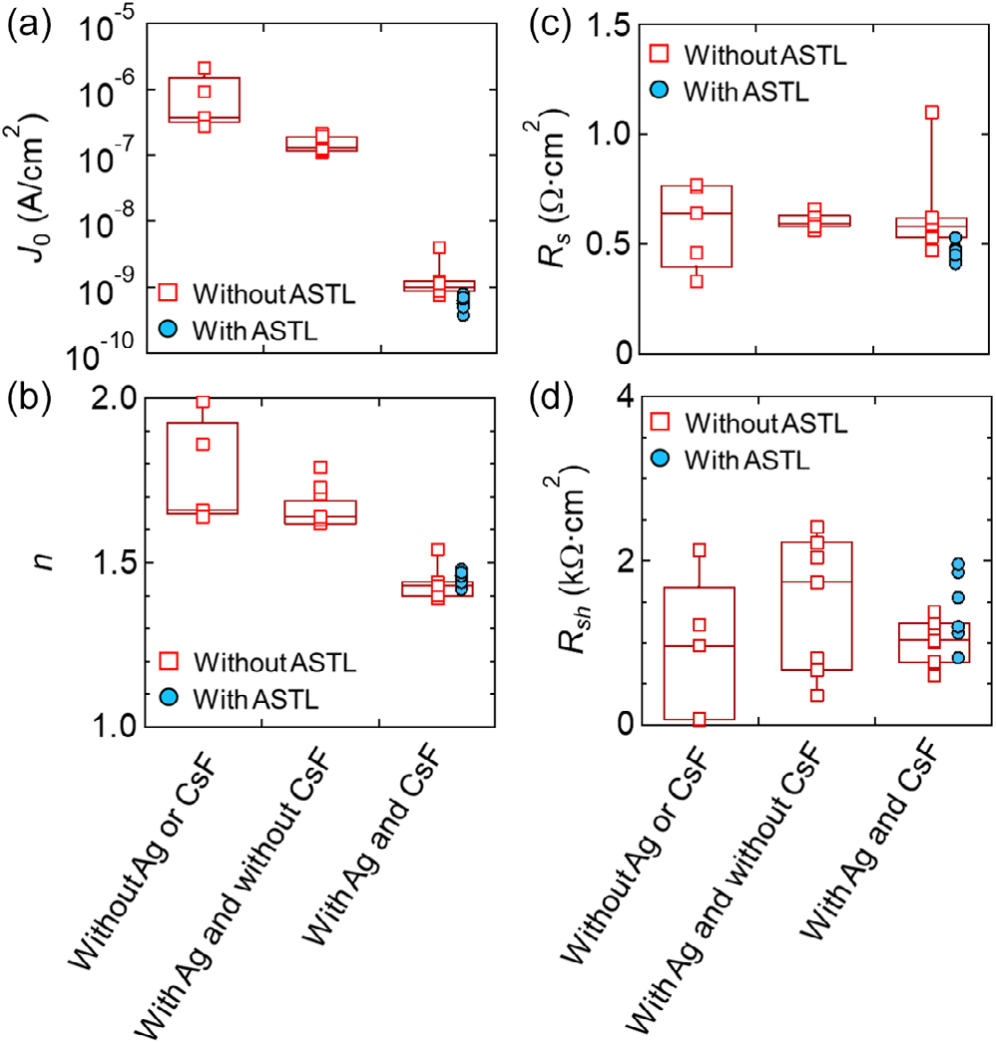
Diode parameters, namely, a) *J*_0_, b) *n*, c) *R*_s_, and d) *R*_sh_, of CIGS solar cells fabricated under three growth conditions: without Ag alloying or CsF‐PDT, with Ag alloying and without CsF‐PDT, and with Ag alloying and CsF‐PDT. The data for the CIGS solar cells without ASTL and with 50 nm thick ASTL are marked as squares and circles, respectively.

To determine why the PV parameters evolved after Ag alloying, the CsF‐PDT, and ASTL deposition, the diode parameters were analyzed. **Figure** **6** shows the diode parameters estimated by analyzing the current density versus bias (*J*–*V*) curves^[^ ^44^ ^]^ in dark, (a) reverse saturation current density (*J*_0_), (b) ideal factor (*n*), (c) series resistance (*R*_s_), and (d) shunt resistance (*R*_sh_) of the CIGS solar cells fabricated under three growth conditions: without Ag alloying or CsF‐PDT, with Ag alloying but without CsF‐PDT, and with Ag alloying and CsF‐PDT. The data for the CIGS solar cells without ASTL and with 50 nm thick ASTL are marked as squares and circles, respectively. Ag alloying primarily reduced *J*_0_, and its impact on *n* was moderate; this indicated reduced carrier recombination in the quasineutral region of the CIGS absorbers. Reduced density of structural and electrical defects by introducing the Ag to low‐temperature growth might be origin of the reduced *J*_0_ and might be origin of improved *V*_OC_ and FF discussed above (Figure 4).^[^ ^32^ ^]^ Meanwhile, the CsF‐PDT remarkably reduced *J*_0_ and *n*, which indicated (at least) reduced carrier recombination in the depletion region. This finding agreed with the previously reported observation that heavy‐alkali‐metal PDT improves the quality of the p–n junction.^[^ ^45^ ^]^ Carrier recombination at the CIGS/CdS interface and its vicinity are expected to improve due to surface bandgap widening and reduced grain‐boundary recombination.^[^ ^28^, ^46^, ^47^ ^]^ The effect of the CsF‐PDT on carrier recombination in the quasi‐neutral region of the CIGS absorbers cannot be determined only from the analysis of diode parameters. However, considering that the CsF‐PDT effectively improved the quality of the grain boundary,^[^ ^28^, ^46^, ^47^ ^]^ reduced recombination would also be expected in the quasi‐neutral region of the CIGS absorbers. Introducing a 50 nm thick ASTL further improved the average *J*_0_ value (albeit slightly) from 1 × 10^−9^ to 6 × 10^−10^ A cm^−2^. As shown in the next subsection, 50 nm is the optimal layer thickness. Introducing the optimal amount of Na can reduce density of detrimental defects such as antisite defects (In_Cu_).^[^ ^48^ ^]^

**Figure 7:**
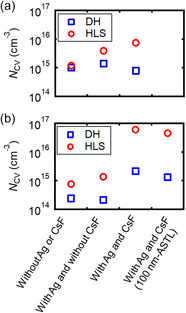
*N*_CV_ of CIGS solar cells fabricated on the a) SLG and b) PI substrates under different conditions: without Ag alloying or CsF‐PDT, with Ag alloying and without CsF‐PDT, with Ag alloying and CsF‐PDT, and with Ag and CsF‐PDT (with 100 nm thick ASTL).

Next, the space charge densities (*N*_CV_) were estimated from the capacitance–voltage (*C*–*V*) measurements (**Figure** **7**)^[^ ^49^ ^]^ of the CIGS solar cells fabricated on (a) SLG and (b) PI substrates under different conditions: without Ag alloying or the CsF‐PDT, with Ag alloying and without the CsF‐PDT, with Ag alloying and the CsF‐PDT, and with Ag and the CsF‐PDT (with 100 nm thick‐ASTL). The characteristics of the CIGS solar cells under relaxed and metastable states were evaluated after dark heating (DH) and heat–light soaking (HLS),^[^ ^50^ ^]^ respectively. The *N*_CV_ values as a function of the space charge width are shown in Figure S3, Supporting Information. With CsF‐PDT and HLS, the *N*_CV_ values increased, indicating an increase in the effective acceptor concentration in the CIGS absorbers. Such an increase was probably caused by an increase in the Cu vacancy (V_Cu_) acceptor and/or a reduction in the detrimental donor defects (antisite defects: In_Cu_ and Ga_Cu_).^[^ ^51^ ^]^

#### 2.1.3 Influence of the SF on Device Performance

**Figure 8:**
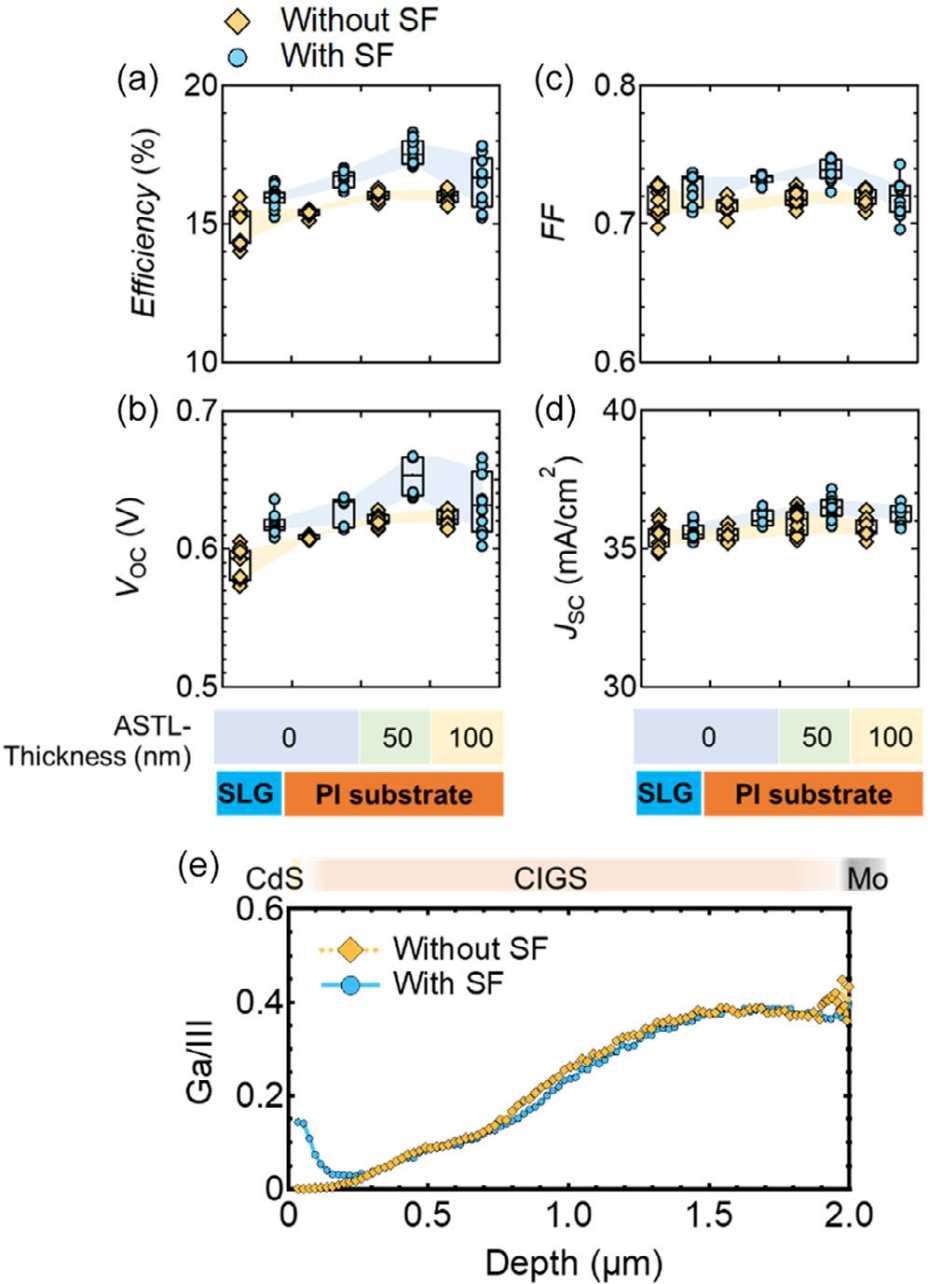
a) Conversion efficiency, b) *V*_OC_, c) FF, and d) *J*_SC_ of CIGS solar cells with and without the SF (denoted as circles and diamonds, respectively) fabricated on the SLG and PI substrates with various ASTL thicknesses (0, 50, and 100 nm) and with Ag alloying and CsF‐PDT. The AR coating was not applied on the devices. e) Ga/III depth profile of the CIGS solar cells with and without the SF (denoted as circles and diamonds, respectively) fabricated on the PI substrates without the ASTL.

**Figure** **8** shows the (a) conversion efficiency, (b) *V*_OC_, (c) FF, and (d) *J*_SC_ of CIGS solar cells with and without the SF (denoted as circles and diamonds, respectively) fabricated on the SLG and PI substrates with various ASTL thicknesses (0, 50, and 100 nm). All samples were fabricated with Ag alloying and the CsF‐PDT. The AR coating was not applied on the devices. (e) Ga/III depth profile of the CIGS solar cells with and without the SF (denoted as circles and diamonds, respectively) fabricated on the PI substrates without the ASTL. Upon introducing the SF, the conversion efficiency of the solar cells under all conditions improved, primarily due to improved *V*_OC_ and FF. This improvement was remarkable in solar cells fabricated on PI substrates without the ASTL and 50‐nm‐thick ASTL. For CIGS solar cells fabricated on the PI substrate coated with a 50‐nm‐thick ASTL, the average *V*_OC_ and FF increased from 0.62 to 0.65 V and from 0.71 to 0.74 by introducing the SF, respectively. The EQE spectra of the samples are shown in Figure S4, Supporting Information. The increase in the short‐wavelength regions might correspond to reduced carrier recombination at the CIGS/CdS interface and/or its vicinity. In contrast, the efficiency gain of CIGS solar cells fabricated on the SLG and PI substrates coated with 100 nm ASTL was moderate.

**Figure 9:**
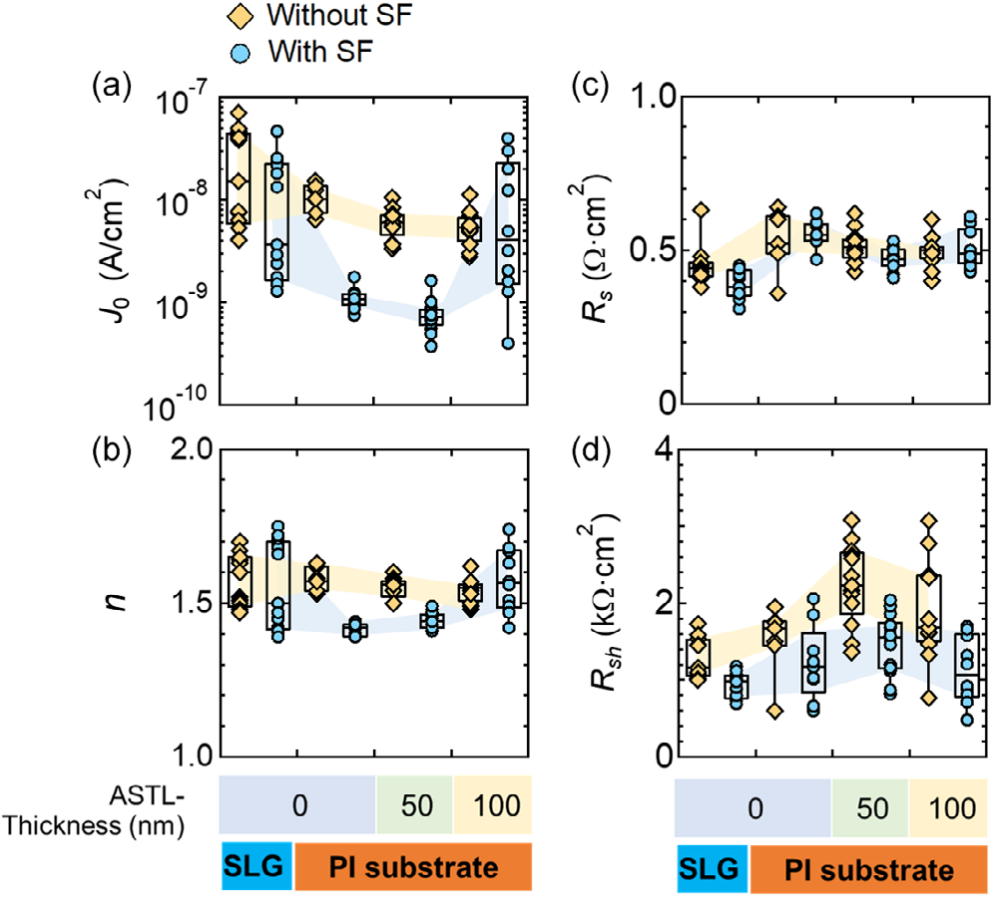
a) *J*_0_, b) *n*, c) *R*_s_, and d) *R*_sh_ of CIGS solar cells with and without the SF (denoted as circles and diamonds, respectively) fabricated on the SLG and PI substrates with different ASTL thicknesses (0, 50, and 100 nm); CsF‐PDT and Ag alloying were performed.

To determine how the PV parameters evolve by introducing SF, the diode parameters were analyzed. **Figure** **9** shows the diode parameters such as (a) *J*_0_, (b) *n*, (c) *R*_s_, and (d) *R*_sh_ of the CIGS solar cells with and without the SF (denoted as circles and diamonds, respectively) fabricated on the SLG and PI substrates with different ASTL thicknesses (0, 50, and 100 nm); the CsF‐PDT and Ag alloying were employed. Upon introducing the SF, the average *J*_0_ and *n* reduced from 6.1 × 10^−9^ to 7.7 × 10^−10^ A cm^−2^ and from 1.55 to 1.44, respectively, for the solar cell fabricated on the PI substrate coated with 50 nm thick ASTL. This indicated (at least) reduced carrier recombination in the space charge region of the CIGS solar cells, which is the presumed origin of the improved *V*_OC_ and FF. In contrast, no significant reduction in *n* was observed for the solar cell fabricated on SLG and PI substrates coated with 100 nm thick ASTL. For the samples prepared on the SLG and PI substrates with 100 nm thick ASTLs, introduction of the SF slightly reduced *J*_0_; however, the reduced *J*_0_ value was still high as ≈4 × 10^9^ A cm^−2^, indicating high carrier recombination in the quasi‐neutral regions. The presence of Na during the growth of CIGS solar cells is known to inhibit CIGS grain growth as well as often decrease the crystal quality of the CIGS layer^[^ ^30^, ^40^, ^41^ ^]^ and excessive Na increases defects such as Na clusters.^[^ ^52^ ^]^ The effect of excessive Na might be more detrimental during low‐temperature growth since the thermal energy available for crystal growth is limited comparing to the case of high‐temperature growth. Therefore, the samples fabricated on the SLG substrate and on the PI substrate with 100 nm thick ASTL were outperformed by samples fabricated on the PI substrate with the optimal ASTL thickness (50 nm). Introducing the optimal amount of Na might reduce density of detrimental defects such as antisite defects (In_Cu_).^[^ ^48^ ^]^ When the quasi‐neutral region contained large number of carrier‐recombination centers, the impact of the SF on the solar cells’ performance was expected to be small. This was because most minority carriers recombined before reaching the SF, as will be discussed in Section 2.2. In contrast, solar cells with improved CIGS bulk quality, such as those fabricated on the PI substrate with optimal Na supply (50 nm thick ASTL), majorly benefited from the SF. The mechanisms of the positive effect of the SF on the solar cells’ performance will be further discussed in detail in Section 2.2.

#### 2.1.4 Champion Device Performance

**Figure 10:**
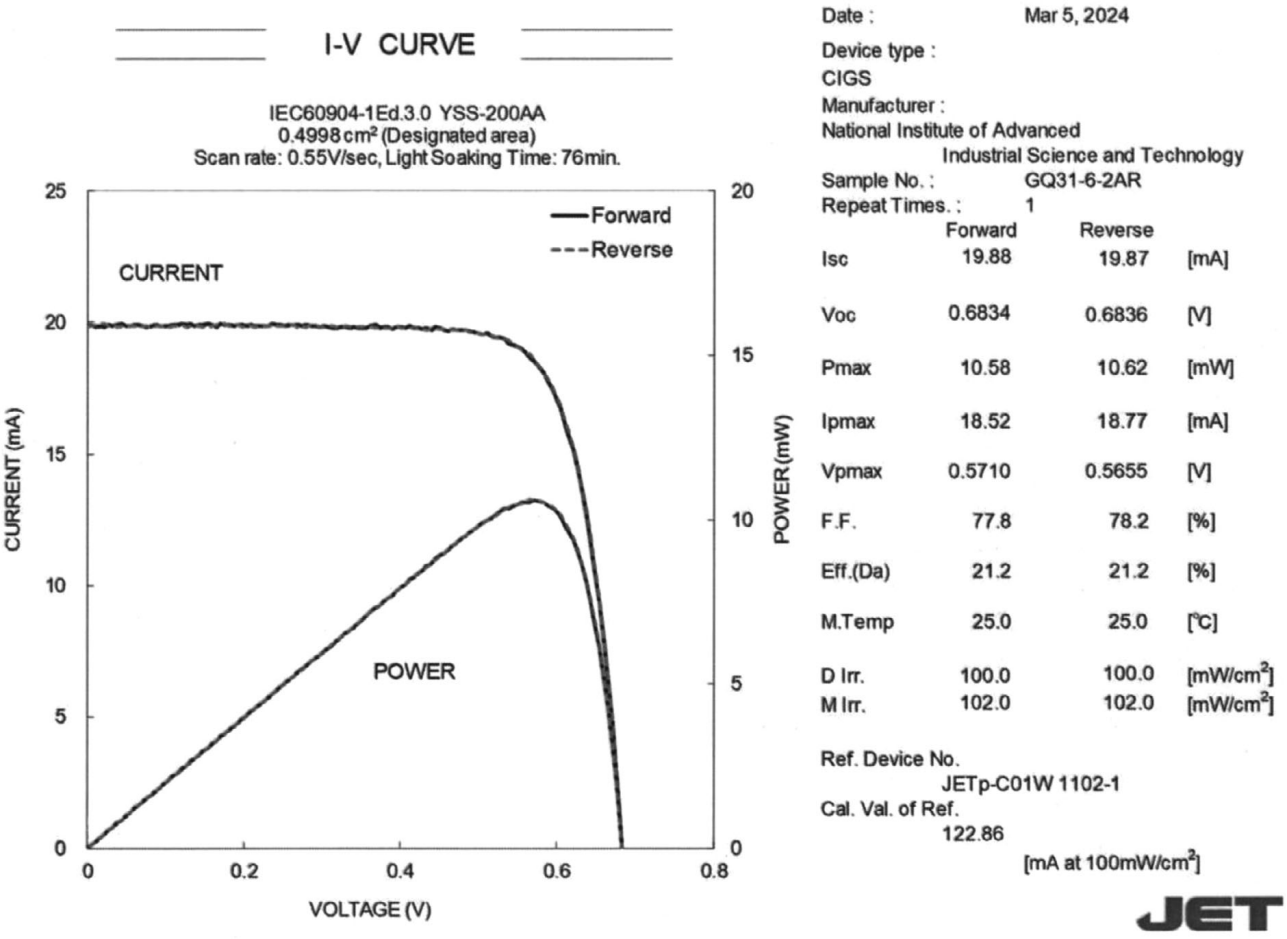
Results of independently certified efficiency measurements of the CIGS solar cell fabricated on the PI substrate coated with a 50 nm thick ASTL with the SF and subjected to Ag alloying and the CsF‐PDT. An AR coating was applied on the device. Photolithography was used for cell separation.

**Figure 11:**
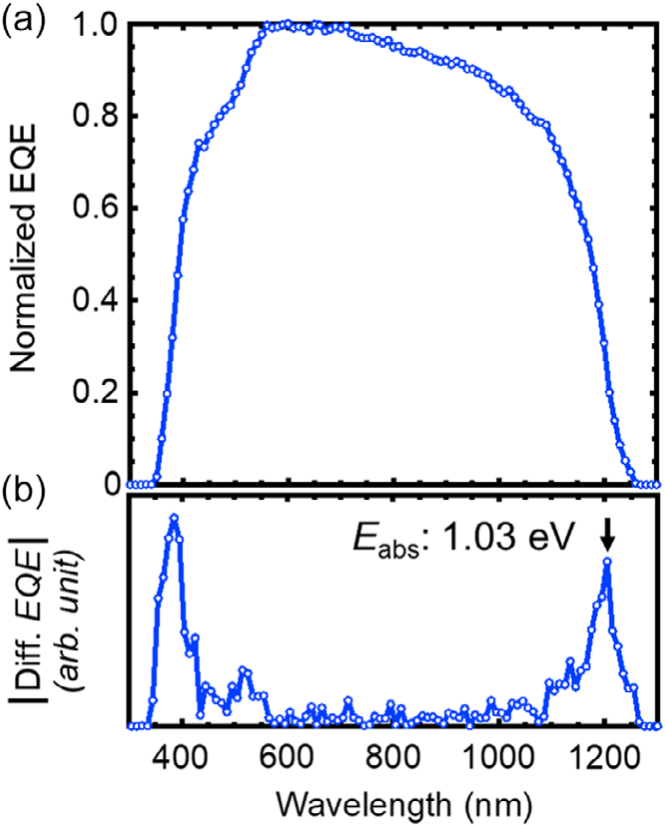
a) Normalized EQE and b) absolute value of the differentiation of the EQE of the CIGS solar cell fabricated on the PI substrate coated with a 50 nm thick ASTL with the SF and subjected to Ag alloying and the CsF‐PDT. An AR coating was applied on the device. Photolithography was used for cell separation.

**Figure** **10** shows the results of independently certified efficiency measurements of the CIGS solar cells fabricated on the PI substrate coated with a 50 nm thick ASTL along with the SF and subjected to Ag alloying and the CsF‐PDT. The PV parameters are listed in **Table** **1**. The normalized and absolute value of differentiation of EQE of the CIGS solar cells are shown in **Figure** **11**.

**Table 1:** PV parameters of the champion CIGS solar cell fabricated on the PI substrate coated with 50‐nm‐thick ASTL.

	Efficiency [%]a)	*V*_OC_ [V]	*I*_SC_ [mA]	*J*_SC_ [mA cm^−2^]	FF	Area [cm^2^]	Test center
Forward scan	21.2	0.6834	19.88	39.78	0.778	0.4998	JET
Reverse scan	21.2	0.6836	19.87	39.76	0.782	0.4998	JET

a) AR coating was applied on the device, and photolithography was used for cell separation.

Photolithography cell separation was employed for the fabricated solar cell.^[^ ^53^ ^]^ An AR coating was applied to the device, and a high conversion efficiency of 21.2% was achieved. The absorption edge of the solar cell, i.e., effective bandgap estimated using the differential of EQE—dEQE/d*λ*, where *λ* is the wavelength—was 1.03 eV. The efficiency was notably high as a CIGS solar cell with narrow bandgap (≈1 eV) fabricated on flexible substrates. *V*_OC_ values of 0.6834 V (forward scan) and 0.6836 V (reverse scan) corresponded to a *V*_OC_ deficit of 0.346 V, which was lower than the *V*_OC_ deficit (0.363 V) reported for the world record CIGS solar cell.^[^ ^10^ ^]^ *J*_SC_ was as high as ≈39.8 mA cm^−2^ owing to the absorption in wide wavelength range up to 1203 nm (*E*_abs_ of ≈1.03 eV). Thus, the solar cell can preferably be used as the bottom cell of tandem solar cells. In addition, FF was ≈0.78, which predominantly improved due to photolithography cell separation,^[^ ^53^ ^]^ thereby improving *R*_sh_. The diode parameters estimated by analyzing the *J*–*V* curve measured in dark are listed in **Table** **2**. Higher *R*_sh_ value, lower *n* value, and lower *J*_0_ value were obtained for the solar cell compared to those of the solar cells subjected to mechanical scribe cell separation (Figure 9) owing to improved edge quality of the former cells.^[^ ^53^ ^]^

**Table 2:** Diode parameters of the champion CIGS solar cell fabricated on the PI substrate coated with 50 nm thick ASTL estimated by analyzing the *J*–*V* curve obtained in dark.

*R*_sh_ [kΩ cm^2^]a)	*R*_s_ [Ω cm^2^]	*N*	*J*_0_ [A cm^−2^]
4.4	0.46	1.34	1.4 × 10^−10^

a) AR coating was applied on the device, and photolithography was used for cell separation.

### 2.2 Investigation of Effects of SF by Device Simulation

#### 2.2.1 SF Introduction and Interfacial Recombination at the Absorber Layer/CdS Interface

**Figure 12:**
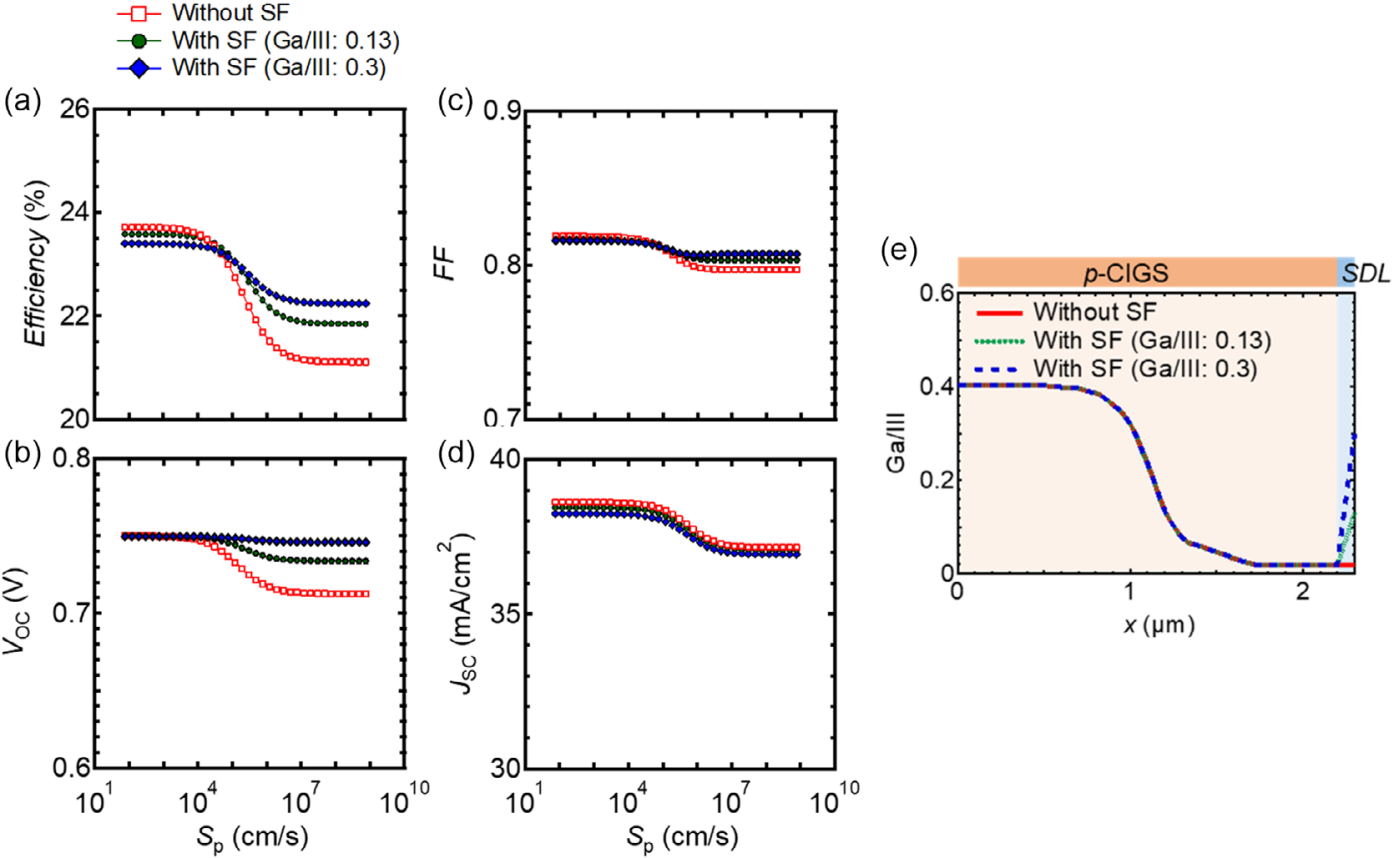
Calculated PV parameters such as a) conversion efficiency, b) *V*_OC_, c) FF, and d) *J*_SC_ using SCAPS for CIGS solar cells with and without the SF as a function of the interfacial recombination velocity of holes (*S*_p_) at the SDL/CdS interface. e) Ga/III depth profiles in the p‐CIGS and SDL regions used for calculations. SDL and CdS were set to have intrinsic and n‐type conductivities (10^17^ cm^−3^), respectively.

Next, the mechanism by which the SF exerts a positive effect on device performances was investigated through device simulations. **Figure** **12** shows the PV parameters such as (a) conversion efficiency, (b) *V*_OC_, (c) FF, and (d) *J*_SC_ calculated using the solar cell capacitance simulator (SCAPS)^[^ ^54^ ^]^ for CIGS solar cells with and without the SF as a function of the interfacial recombination velocity of holes (*S*_p_) at the absorber layer/CdS interface from absorber layer side. The Cu‐deficient region at the surface of the CIGS absorber layer^[^ ^55^ ^]^ was defined as the surface defect layer (SDL).^[^ ^56^ ^]^ The interfacial recombination velocity at the SDL/CdS interface was varied by changing the density of neutral defects (*N*_t_) located at the center of the interface gap. *N*_t_ varied from 10^10^ to 10^17^ cm^−2^, corresponding to the variation in the *S*_p_ from 75 to 7.5 × 10^8^ cm s^−1^. For simplicity, no defects were introduced to the SDL. In the calculation, the conductivity of SDL and CdS was set as intrinsic and n‐type (10^17^ cm^−3^), respectively. Figure 12e shows the Ga/III depth profiles of the p‐CIGS and SDL regions used in the SCAPS calculations, and the typical input parameters are listed in **Table** **3**. Here, the *L*_n_ values of the CIGS and SDL regions were set to large values (5 μm and ∞) to focus on the influences of recombination at the SDL/CdS interface on the device performance. Without the SF, the PV parameters decreased with increasing *S*_p_. In contrast, with the SF, the decrease in *V*_OC_ was mitigated. Similar trends were observed in FF although its variation was lower. The influence of the SF on *J*_SC_ was small under all the calculation conditions. The decrease in the PV parameters for the case without the SF became notable when *S*_p_ became >10^4^ cm s^−1^ under the calculation condition. However, note that the *S*_p_ values presented in Figure 12 cannot be quantitatively compared directly with the experimental results as the calculation included many simplifications. For example, the defects in the SDL, which we neglected in the calculation, could increase the impact of the SF.

**Table 3:** Material parameters of p‐CIGS, SDL, CdS, i‐ZnO, and n‐ZnO used for SCAPS calculation.

	p‐CIGS	SDL	CdS	i*‐*ZnO	n*‐*ZnO
Thickness [μm]	2.2	0.1	0.045	0.05	0.3
Dielectric constant [C V^−1^ m^−1^]	13.6 × *ε*_0_	13.6 × *ε*_0_	10 × *ε*_0_	9 × *ε*_0_	9 × *ε*_0_
Effective mass of electrons	0.2 × *m*_0_	0.2 × *m*_0_	0.2 × *m*_0_	0.2 × *m*_0_	0.2 × *m*_0_
Effective mass of holes	0.8 × *m*_0_	0.8 × *m*_0_	0.8 × *m*_0_	0.8 × *m*_0_	0.8 × *m*_0_
Mobility of electrons [cm V^−1^ s^−1^]	40	40	100	180	180
Mobility of holes [cm V^−1^ s^−1^]	10	10	25	45	45
Electron affinity [eV]	4.35 − 0.459*y *− 0.167 *y* ^2^	4.37 − 0.415*y *− 0.24 *y* ^2^	4.05	4.15	4.15
Bandgap [eV]	1.01 + 0.459*y *+ 0.167 *y* ^2^	1.193 + 0.415*y* + 0.24 *y* ^2^	2.4	3.3	3.3
Carrier density [cm^−3^]	2 × 10^16^	Variable	Variable	1 × 10^17^	4 × 10^20^

**Figure 13:**
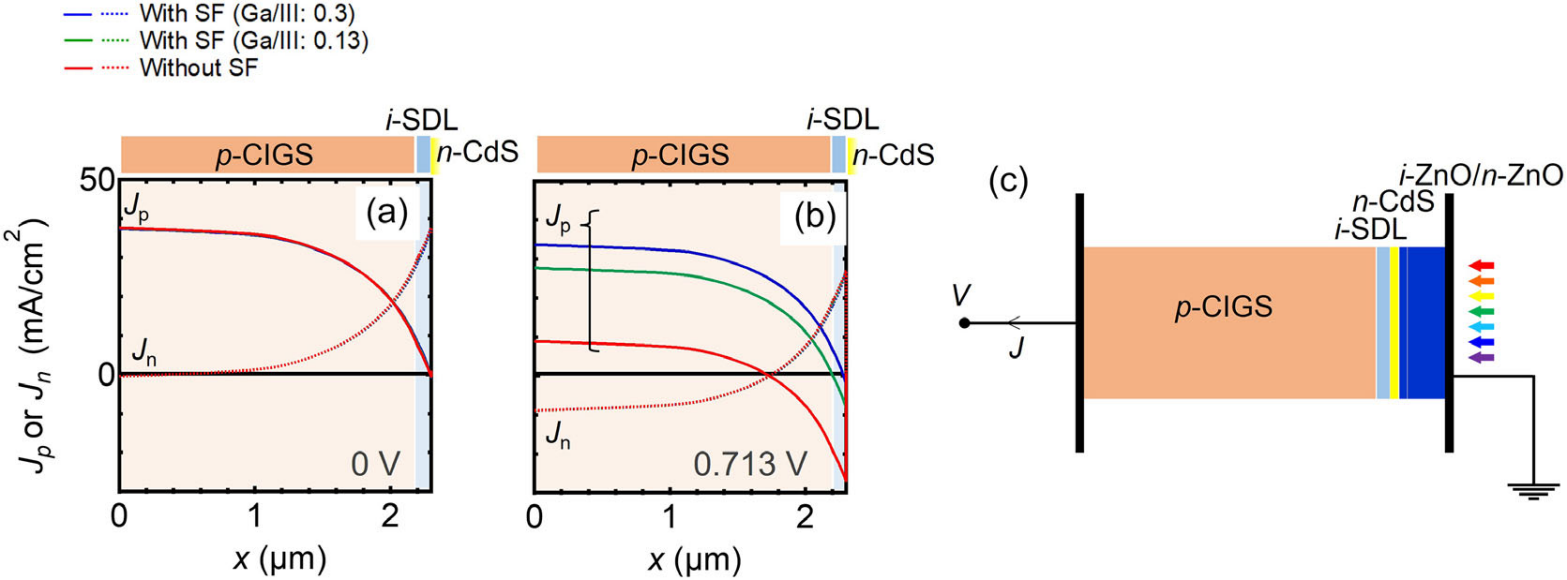
Calculated electron current density (*J*_e_) and hole current density (*J*_h_) distribution in CIGS solar cells with and without the SF at biases (*V*_Bias_) of a) 0 V and b) 0.713 V. c) The layer structure used for calculations.

**Figure** **13** shows the calculated electron current density (*J*_e_) and hole current density (*J*_h_) distribution in CIGS solar cells with and without the SF at the bias conditions of (a) 0 V and (b) 0.713 V which is the *V*_OC_ of the solar cell without the SF. The total defect density at the SDL/CdS interface was set to 1.35 × 10^15^ cm^−2^, corresponding to an *S*_p_ of 10^7^ cm s^−1^, as an extreme case of interfacial recombination. Figure 13c shows the layer structure used for calculations; the current density *J* (*J*_h_ + *J*_e_) in the photocurrent direction, i.e., current flow from the n side to p side, was defined as positive. The difference in *J*_e_ and *J*_h_ between the CIGS solar cells with and without the SF was not large at 0 V. At 0.713 V, *J*_h_ reduced remarkably as the SF was introduced. This indicated that the hole diode current flowing from p‐CIGS to the SDL/CdS interface was reduced after the introduction of the SF. No large difference was observed in *J*_e_ after the introduction of the SF.

**Figure 14:**
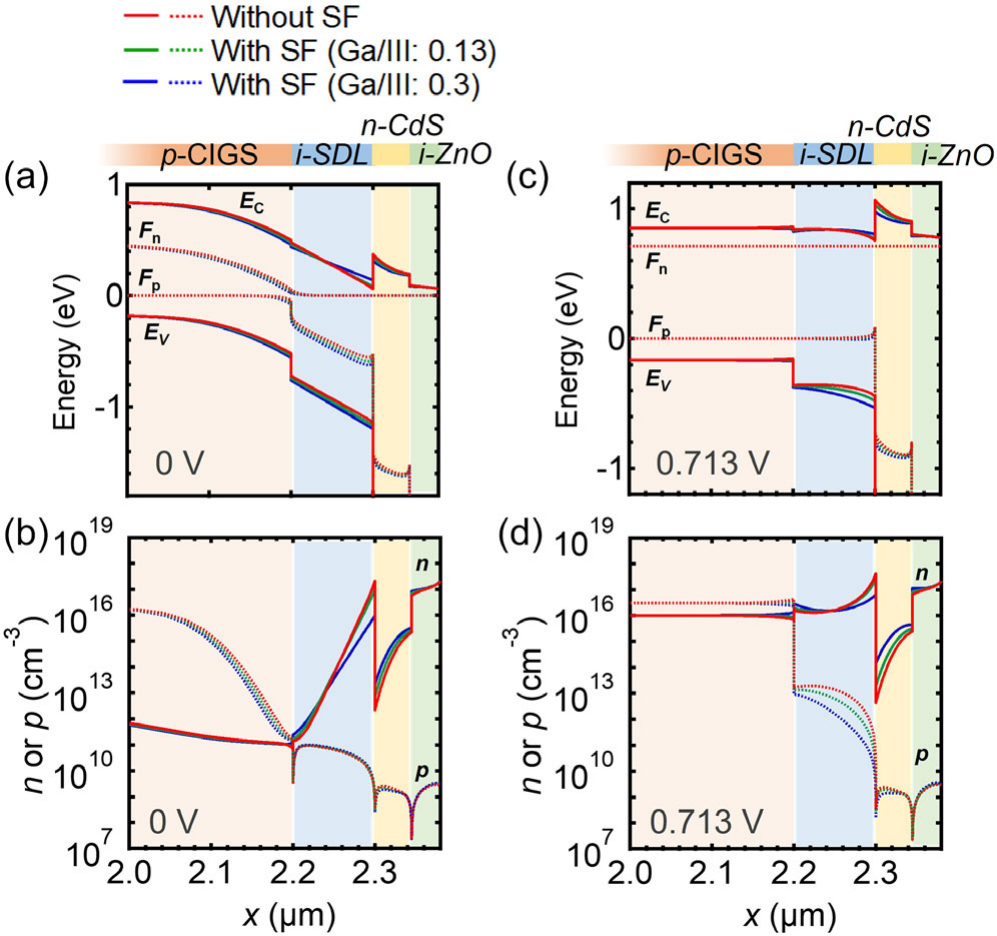
Calculated band diagrams and distribution of electron density (n) and hole density (p) at a,b) 0 V and c,d) 0.713 V.

**Figure** **14** shows the band diagrams and distributions of electron density (n) and hole density (p) at (a, b) 0 V and (c, d) 0.713 V. *E*_C_, *E*_V_, *F*_n_, and *F*_p_ are the energy of bottom of the conduction band, top of the valence band, and quasi‐Fermi energy for electrons and holes, respectively. At the bias condition close to *V*_OC_, the SF formed by an increase in Ga/III toward the SDL/CdS interface caused a downward shift in *E*_V_ compared with the CIGS solar cells without the SF. An increase in Ga/III resulted in a downward shift in *E*_V_ could sound unusual because increased Ga/III mainly reduced the electron affinity and thus caused an upward shift in *E*_C_ in the neutral region. Under the forward bias condition (≈*V*_OC_) in the depletion region, electrons were injected from the n‐CdS side, thereby increasing the electron density and hence the *F*_n_. The energy difference between *E*_C_ and *F*_n_ is related to electron density (n) as follows:

(1) $$F_{n}(x)-E_{C}(x)=\zeta_{n}(x)$$

where $\zeta_{n}$ is the chemical potential of electrons.

(2) $$\zeta_{n}(x)=k_{B}T\ln\left(\frac{n(x)}{N_{C}}\right)$$

which is a function of the electron density {*n*(*x*)}. *N*_C_ is the effective density of states of the conduction band, which was set to 2.2 × 10^18^ cm^−3^ for the CIGS solar cells and the SDL in the calculations. Electron injection caused an increase in $\zeta_{n}\left(x\right)$. As $\zeta_{n}\left(x\right)$ has a negative value, the absolute value of $\left|\zeta_{n}(x)\right|$, i.e., the energy difference between *E*_C_ and *F*_n_, decreased, indicating that *F*_n_ moved closer to *E*_C_. Under the *V*_OC_ condition, *F*_n_ became almost flat throughout the depletion region (Figure 14). Thus, an increase in *E*_g_ with increasing Ga/III resulted in the variation of *E*_V_ in the depletion region as the energy difference between *E*_C_ and *F*_n_ decreased. In other words, introducing the SF increases the electrical field under the near‐*V*_OC_ bias condition in the depletion region of the CIGS absorber (SDL in the calculation). The influence of electron injection from the CdS side was more pronounced when the SDL and CdS exhibited n‐type conductivity (Figure S5 and S6, Supporting Information). In contrast, the influence of electron injection was smaller when the SDL and CdS exhibited p‐type and intrinsic conductivities, respectively (Figure S7 and S8, Supporting Information). Although the magnitude of the effect differs, in all investigated cases, efficiency gains owing to the increased electrical field were expected. The resulting downward shift in *E*_V_ created effective barriers that repulsed holes injected toward the SDL/CdS interface from p‐CIGS, improving the diode quality.

Moreover, when the *S*_p_ is low (e.g., ≈10^3^ cm s^−1^ at the CIS/CdS interface^[^ ^57^ ^]^), no efficiency gain is expected by SF introduction (Figure 12). In fact, under low *S*_p_ conditions, the expected conversion efficiency was rather slightly higher in the sample without the SF than in the sample with the SF (Figure 12). On the other hand, introducing the SF improved the efficiency of the present samples fabricated via low‐temperature growth (Section 2.1.3).

It indicates the *S*_p_ at the CIGS/CdS interface and/or its vicinity is not low enough in our present samples probably owing to low‐temperature growth. There could be rooms for further improvement of the quality of the CIGS/CdS interface and/or its vicinity by optimizing the conditions of Ag alloying and the alkali‐metal PDT. In future research, the quality of the interface and/or CIGS surface region should be sufficiently increased to achieve high efficiency without the SF.

#### 2.2.2 Introduction of SF and *L*_n_ in the CIGS Layer

**Figure 15:**
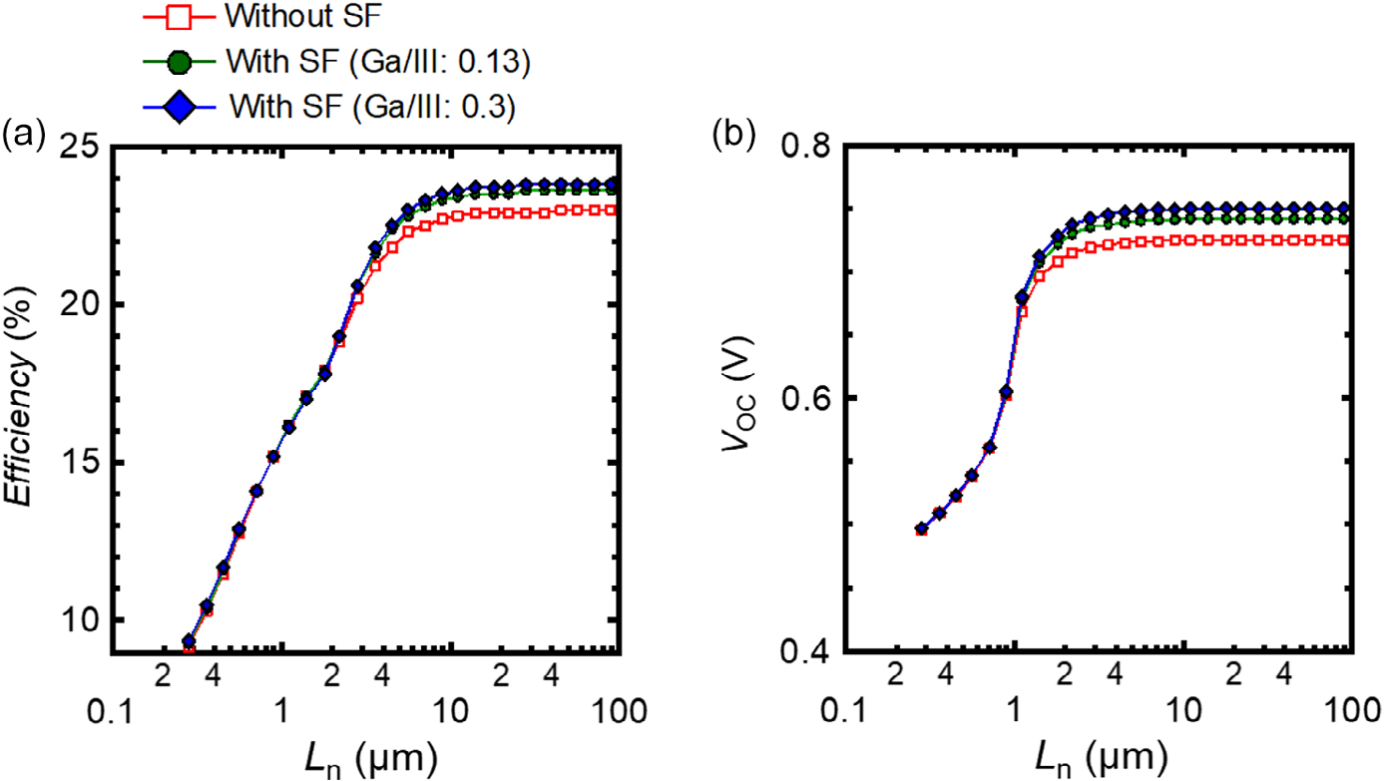
Calculated PV parameters, namely, a) conversion efficiency and b) *V*_OC_ of CIGS solar cells with and without the SF as a function of the minority carrier diffusion length (*L*_n_) in the CIGS layer.

Next, the influence of the *L*_n_ of the CIGS layer on the positive effect of the SF on device performances was studied. **Figure** **15** shows the calculated PV parameters, i.e., (a) conversion efficiency and (b) *V*_OC_ of CIGS solar cells with and without the SF as a function of *L*_n_ in the CIGS layer. The *S*_p_ at the SDL/CdS interface was set to a constant of 10^7^ cm s^−1^. In the calculation, the conductivity of SDL and CdS was set as intrinsic and n‐type (10^17^ cm^−3^), respectively. The efficiency gains mainly resulting from increased *V*_OC_ were obtained when *L*_n_ > 1 μm, i.e., close to or more than the thickness of the CIGS layer (≈2 μm). In contrast, when *L*_n_ was reduced to the order of submicrometer scale, efficiency gain was not achieved even after the introduction of the SF. This was because the predominant recombination process changed from interfacial recombination to bulk recombination. These findings clearly indicated that the high crystal quality of the absorber layer is the prerequisite for exploiting the SF. The experimental results discussed in Section 2.1 showed that our champion device—the CIGS solar cell fabricated with optimum Na supply (50 nm thick ASTL), Ag alloying, and the CsF‐PDT as well as containing the SF—met the requirements.

## 3 Conclusions

In this study, lightweight, flexible CIGS solar cells with a narrow energy bandgap of ≈1.03 eV were fabricated on PI substrates under different growth conditions. CIGS solar cells fabricated at a low growth temperature of ≈400 °C suffer from low performance, which was considerably improved by applying several methods. Ag alloying and light‐alkali‐metal (Na) doping via ASTL deposition improved the quality of the CIGS bulk, and heavy‐alkali‐metal postdeposition treatment (CsF‐PDT) improved the interfacial quality. Moreover, front shallow Ga grading, i.e., the SF, was introduced to reduce carrier recombination at the CIGS/CdS interface and/or its vicinity. This SF effectively improved the performance of CIGS solar cells fabricated on PI substrates without ASTL and coated with 50 nm thick ASTL, i.e., without Na and with moderate Na supply during the growth of CIGS solar cells. In contrast, solar cells fabricated on the SLG and PI substrate coated with a 100 nm thick ASTL were not highly benefited from the SF. Device simulation revealed that low carrier recombination in the quasineutral region, e.g., in the CIGS bulk, is prerequisite to benefit from the SF. When carrier recombination in the quasineutral region was large, i.e., *L*_n_ was small, the impact of SF was small. This was because the majority of minority carriers recombined before reaching the SF. Thus, the CIGS solar cells with enhanced CIGS bulk quality, such as those fabricated on PI substrates coated with a 50 nm thick ASTL and subjected to Ag alloying and CsF‐PDT considerably benefited from the inclusion of the SF. The introduction of the SF increased the electrical field in the depletion region of the CIGS absorber. The champion CIGS solar cell fabricated via Ag alloying, the CsF‐PDT, photolithography cell separation, and AR coating with an optimum Na supply and the SF exhibited a notably high conversion efficiency of 21.2%, low *V*_OC_ deficit of 0.346 V, and high *J*_SC_ of ≈40 mA cm^−2^.

## 4 Experimental Section

4.1

4.1.1

##### Fabrication and Evaluation of CIGS Layer and CIGS Solar Cells

PI varnish UPIA,^[^ ^25^ ^]^ which is polyamic acid precursor solution of PI, was coated on the glass support via spin coating. The precursor films were then subjected to thermal annealing in dry N_2_ up to 420 °C to form PI films, which were then subjected to annealing up to 420 °C in a vacuum chamber to eliminate possible outgassing. Subsequently, the Mo back contact layer was sputtered without intentional heating via in‐line‐type direct current (DC) sputtering. A 0.2 μm thick Mo layer was deposited on the PI substrate at an Ar pressure (*P*_Ar_) of 0.1 Pa; the same layer was deposited 4 times (total thickness: 0.8 μm) on reference SLG substrates at a *P*_Ar_ of 0.5 Pa. The ASTL,^[^ ^24^ ^]^ which acted as an alkaline metal source, was deposited on some PI substrates via radio frequency (RF) sputtering before depositing the Mo layer. An ASTL does not change the flexibility of PI substrates.^[^ ^58^ ^]^ In this work, the Na dopant was sourced from the ASTL alone and was not contributed by PDT. The Ag precursor layer of ≈10 nm thickness, as evaluated by a crystal oscillator, was deposited on the Mo layer in the same vacuum chamber used for fabricating CIGS absorbers. Typically, the Ag content was ≈0.2%, as evaluated using an electron probe microanalyzer at 5 kV. As shown in Figure S9, Supporting Information, the Ag content remained relatively constant throughout the CIGS absorber layer along the depth direction. Then, CIGS absorber layers were grown on the Mo back contact layer with and without Ag layer via coevaporation.^[^ ^15^, ^59^ ^]^ The substrate temperatures for stages I, II, and III were set to 350, 400, and 400 °C, respectively. For the fabrication of the samples with the SF, the stage III was divided into two parts (i.e., the stage III‐A followed by stage III‐B). In the stage III‐A, In and Se fluxes were supplied. In the stage III‐B, In, Ga, and Se fluxes are supplied. By precisely controlling molecular beam fluxes and deposition times, the average Cu/(In + Ga) ratio was controlled to be ≈0.93. A CsF layer of ≈10 nm thickness, as evaluated using a crystal oscillator, was deposited on the CIGS absorber layer of some samples under a Se atmosphere in the vacuum chamber used for the CIGS deposition. The substrate temperature was 350 °C and the typical deposition duration was less than 10 min. A CdS buffer layer of ≈45 nm thickness was deposited on the CIGS absorber layer via chemical bath deposition.^[^ ^60^ ^]^ Intrinsic ZnO (i‐ZnO) and n‐type ZnO:Al (n‐ZnO) layers were deposited on the CdS layer via RF and DC sputtering, respectively. The i‐ZnO and n‐ZnO layers were ≈0.06 and 0.35 μm thick, respectively. Finally, an Al front electrode was formed on the n‐ZnO layer via electron beam‐physical vapor deposition. Eight solar cells with a size of ≈0.64 cm^2^ were fabricated in a 3 cm × 3 cm sample via cell separation using mechanical scribing. Photolithography cell separation based on ZnO layer removal using dilute HCl solution was employed to define the cell area as ≈0.5 cm^2^ for the champion solar cell.^[^ ^53^ ^]^ Subsequently, 110 nm thick MgF_2_ AR coating was deposited on the solar cell via thermal evaporation. Unless otherwise stated, HLS, i.e., white‐light irradiation, was performed at 95 °C^[^ ^50^ ^]^ before evaluating the solar cells for stabilizing their PV and electrical properties for 24 h. Some samples underwent DH, i.e., annealing in dark condition at 95 °C, for 24 h only. Current–voltage (*C*–*V)* measurements were conducted at a frequency of 100 kHz using an inductance–capacitance–resistance meter (Agilent E4980A). The PV properties of the solar cells were evaluated via current density–voltage (*J*–*V*) measurements under 100 mW cm^−2^ illumination with a Xe lamp at 25 °C calibrated with a Si photodetector. The evaluated *J*_SC_ and conversion efficiency measured in house were downward corrected using the estimated *J*_SC_ from the EQE spectra to avoid overestimating the conversion efficiency. SIMS measurements were conducted using Cs^+^ or O_2_+ as the primary ions to measure alkali‐metal and constituent element concentrations as a function of distance from the CIGS solar cell surface.

##### Device Simulation

1D device modeling was performed by solving the Poisson equation coupled with the electron and hole continuity equations to determine the electric potential, electron concentration, n, and hole concentration, p, as the functions of space using the SCAPS‐1D software.^[^ ^54^ ^]^ The typical modeled CIGS device structure was front contact/n‐ZnO/i‐ZnO/n‐CdS/i‐SDL/p*‐*CIGS/back contact, as shown in Figure 13 (c). The corresponding input parameters are listed in Table 3, which are based largely on previously reported values.^[^ ^56^, ^61^, ^62^ ^]^ The absorption coefficient of CIGS was based on Hara et al.'s study.^[^ ^43^ ^]^ The interfacial recombination velocity was varied by changing the density of *N*_t_ located in the middle of the interface gap. *N*_t_ was varied from 10^10^ to 10^17^ cm^−2^, corresponding to the *S*_p_ from 75 to 7.5 × 10^8^ cm s^−1^. The bandgap and electrical affinity were set as the functions of Ga/III (*y* in Table 3). Figure 12e shows the depth profiles of Ga/III in the p*‐*CIGS and SDL regions used as the input data for SCAPS calculations. Ga/III in the CIGS region (0–2.2 μm) was set to a single graded structure. Three types of Ga/III gradings in the SDL region (2.2–2.3 μm), i.e., 1) constant values of 0.02, 2) linearly increasing up to 0.13, and 3) 0.3 toward the SDL/CdS boundary, were studied. Case (2) was similar to the experimental SIMS data (Figure 3), while case (3) involved a steeper Ga/III grading. A donor‐type defect was introduced into the CIGS absorber layer at 0.55 eV above the valence band (*E*_V_). Unless clearly stated, the density of the defects in the CIGS absorber layer was set to 3.2 × 10^11^ cm^−3^, corresponding to the *L*_n_ of 5 μm. Moreover, no defects were introduced into the SDL layer. The capture cross sections were set as 5 × 10^−13^ and 1 × 10^−15^ cm^2^ for electrons and holes, respectively, and the recombination velocity of electrons and holes at the back contact was set to 10^5^ cm s^−1^. The recombination velocity at the SDL/CdS interface was varied by changing the density of neutral defects (*N*_t_) at the center of the interface gap from 10^10^ to 10^17^ cm^−2^, thus varying the *S*_p_ from 75 to 7.5 × 10^8^ cm s^−1^.

## Conflict of Interest

The authors declare no conflict of interest.

## Author Contributions

**Yukiko Kamikawa**: conceptualization (lead); data curation (lead); funding acquisition (lead); investigation (lead); methodology (lead); visualization (lead); writing—original draft (lead); writing—review and editing (lead). **Jiro Nishinaga**: methodology (supporting). **Takeshi Nishida**: methodology (supporting). **Shogo Ishizuka**: data curation (supporting); funding acquisition (lead); methodology (supporting); project administration (lead).

## Supporting information

Supplementary Material

## Data Availability

The data that support the findings of this study are available from the corresponding author upon reasonable request.
