# Supplementary material for: Highly Efficient Lightweight Flexible Cu(In,Ga)Se2 Solar Cells with a Narrow Bandgap Fabricated on Polyimide Substrates: Impact of Ag Alloying, Cs and Na Doping, and Front Shallow Ga Grading on Cell Performance
Source: Small Sci. 2024 Dec 16;5(2):2400404. doi: 10.1002/smsc.202400404 (PMC11934904; doi:10.1002/smsc.202400404)
Supplement: Supplementary file 1 — Supplementary Material [file SMSC-5-2400404-s001.pdf]

Supporting Information

Highly efficient lightweight flexible Cu(In,Ga)Se_2 solar cells with a narrow bandgap fabricated on polyimide substrates: impact of Ag alloying, Cs and Na doping, and front shallow Ga grading on cell performance

Yukiko Kamikawa, Jiro Nishinaga, Takeshi Nishida, Shogo Ishizuka*

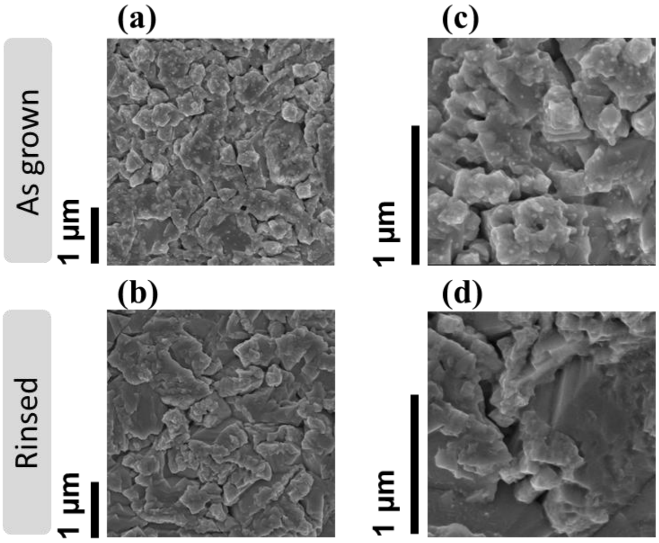

Figure S1 Cross-sectional SEM images of the (a, c) as-grown CIGS absorbers on Mo-coated SLG with Ag and CsF-PDT and (b, d) after rinsing with water.

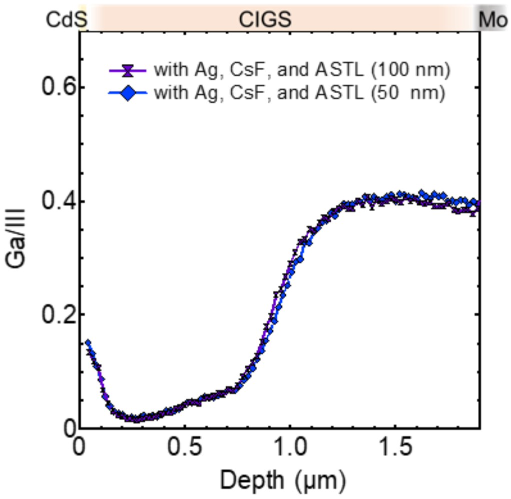

Figure S2. Ga/III in the CIGS layer fabricated with Ag alloying and CsF-PDT on Mo-coated and 50-nm-thick and 100-nm-thick ASTL-coated PI substrates evaluated using SIMS.

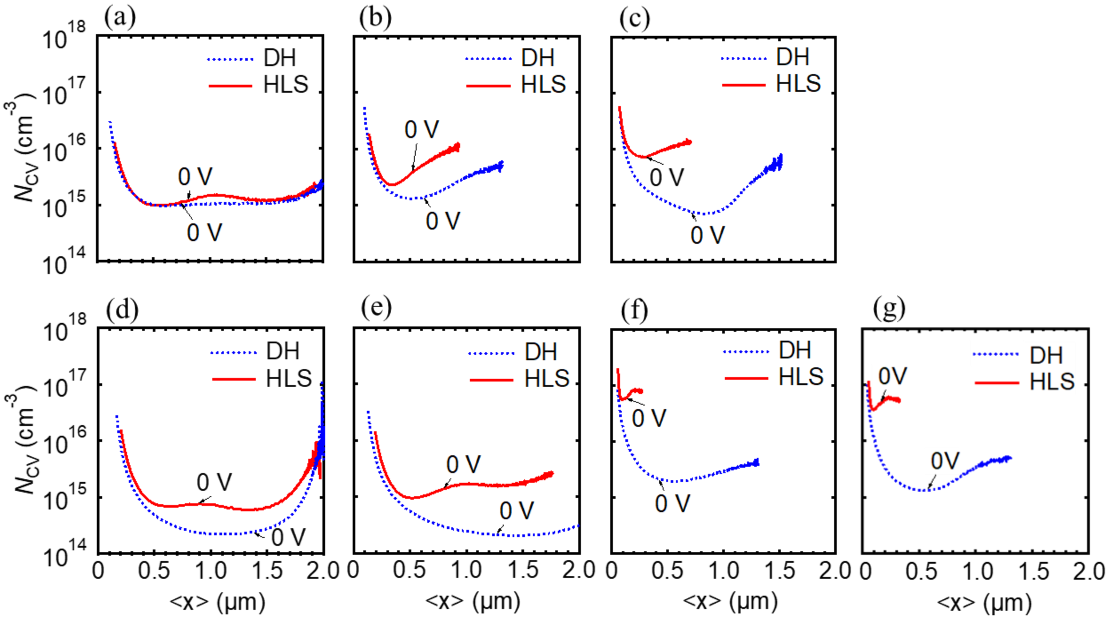

Figure S3. N_{CV} as a function of space charge width <*x*> for CIGS solar cells fabricated (a, d) without Ag alloying or CsF-PDT, (b, e) with Ag alloying and without CsF-PDT, (c, f, g) with Ag alloying and CsF-PDT after HLS or DH. (a–c) are the data for samples fabricated on the SLG substrates and (d–f) are the data for those fabricated on PI substrates without the ASTL layer. (g) Data for the sample deposited on the PI substrate coated with 100-nm-thick ASTL.

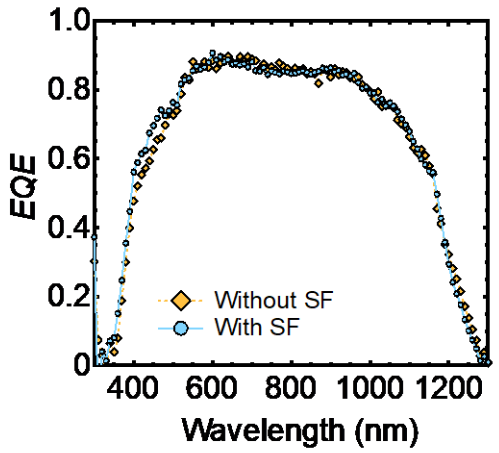

Figure S4. EQE spectra of CIGS solar cells fabricated on the PI substrate coated with 50-nm-thick ASTL with and without the SF. CsF-PDT and Ag alloying were both applied.

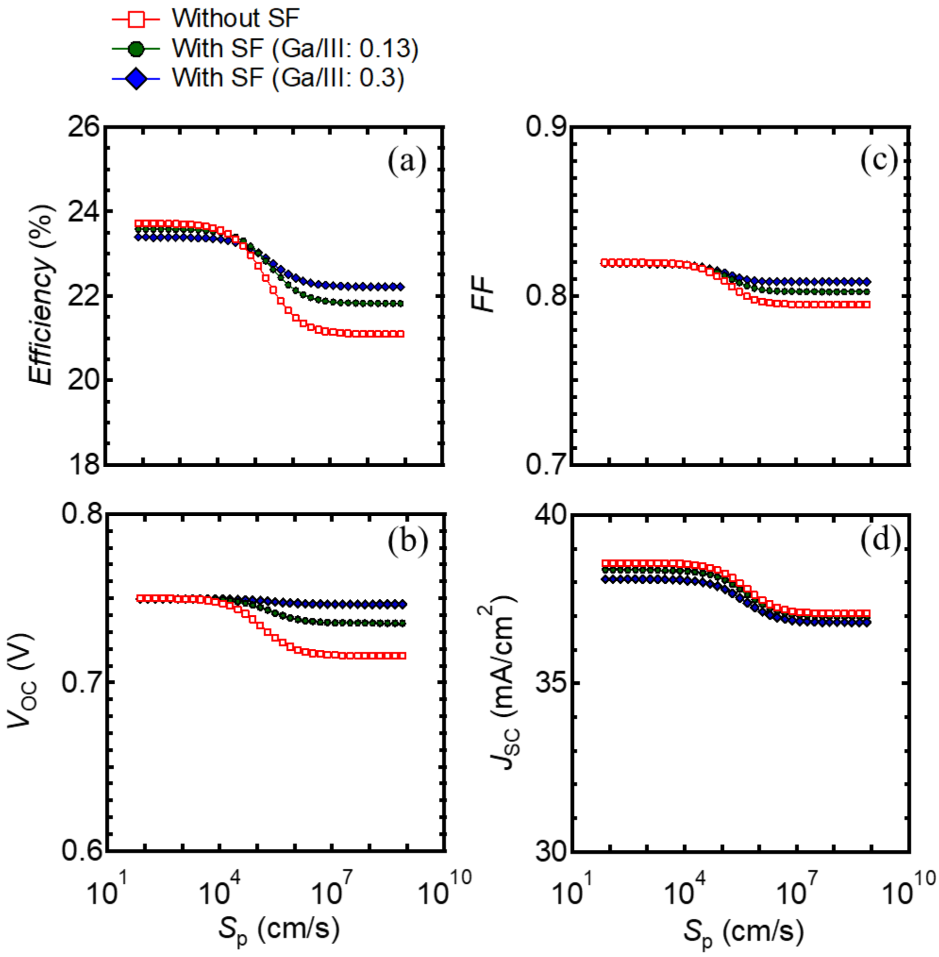

Figure S5. Calculated photovoltaic parameters, i.e., (a) conversion efficiency, (b) V_{OC}, (c) *FF*, (d) J_{SC} using SCAPS for CIGS solar cells with and without the SF. In the calculation the conductivity of SDL and CdS were both defined as *n*-type of 10^{16} and 10^{17} cm^{-3}, respectively.

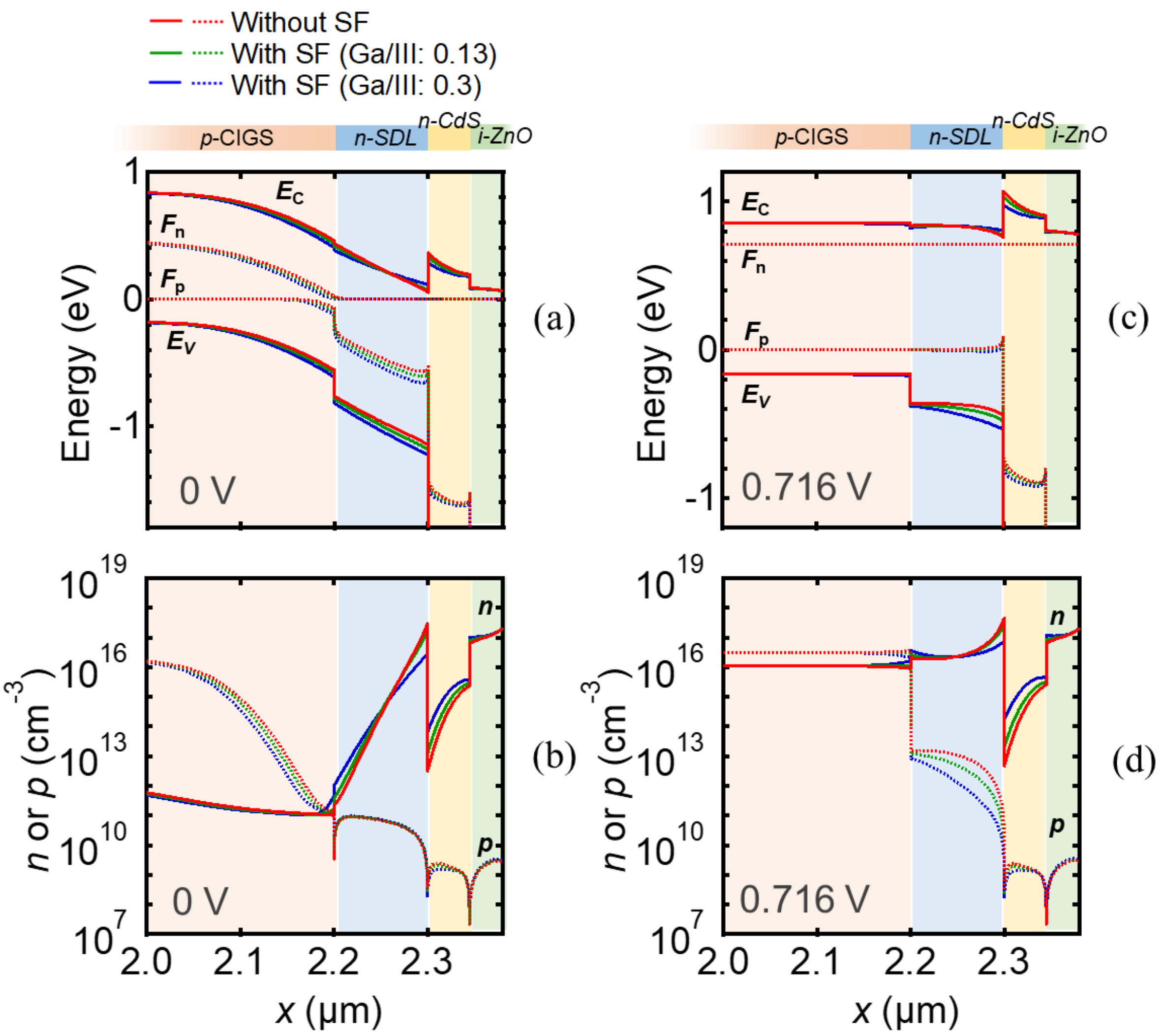

Figure S6. Calculated band diagrams and distribution of electron density (n) and hole density (p) at (a, b) 0 V and (c, d) 0.716 V using SCAPS for CIGS solar cells with and without the SF. In the calculation the conductivity of SDL and CdS were both defined as n-type of 10^{16} and 10^{17} cm^{-3}, respectively.

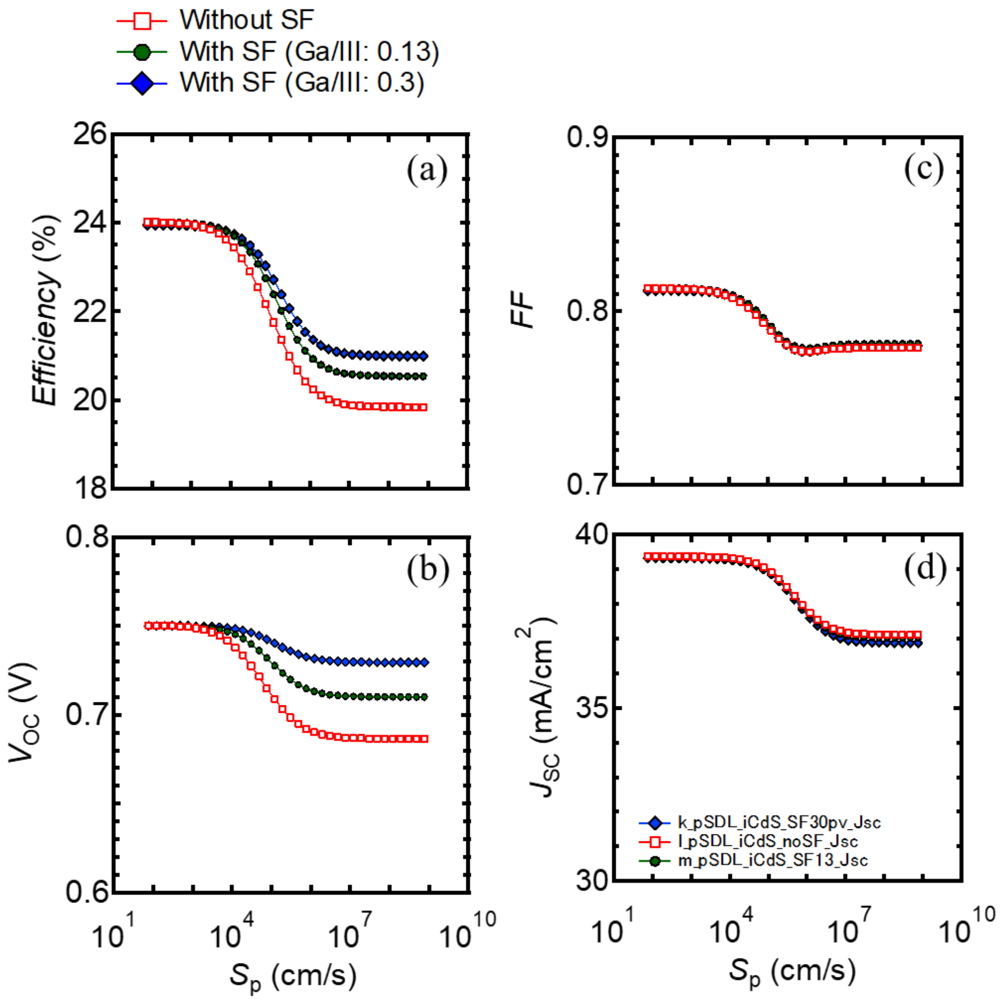

Figure S7. Calculated photovoltaic parameters, i.e., (a) conversion efficiency, (b) V_{OC}, (c) *FF*, and (d) J_{SC} using SCAPS for CIGS solar cells with and without the SF. In the calculation the conductivity of SDL and CdS were defined as *p*-type (10^{16} cm^{-3}) and intrinsic, respectively.

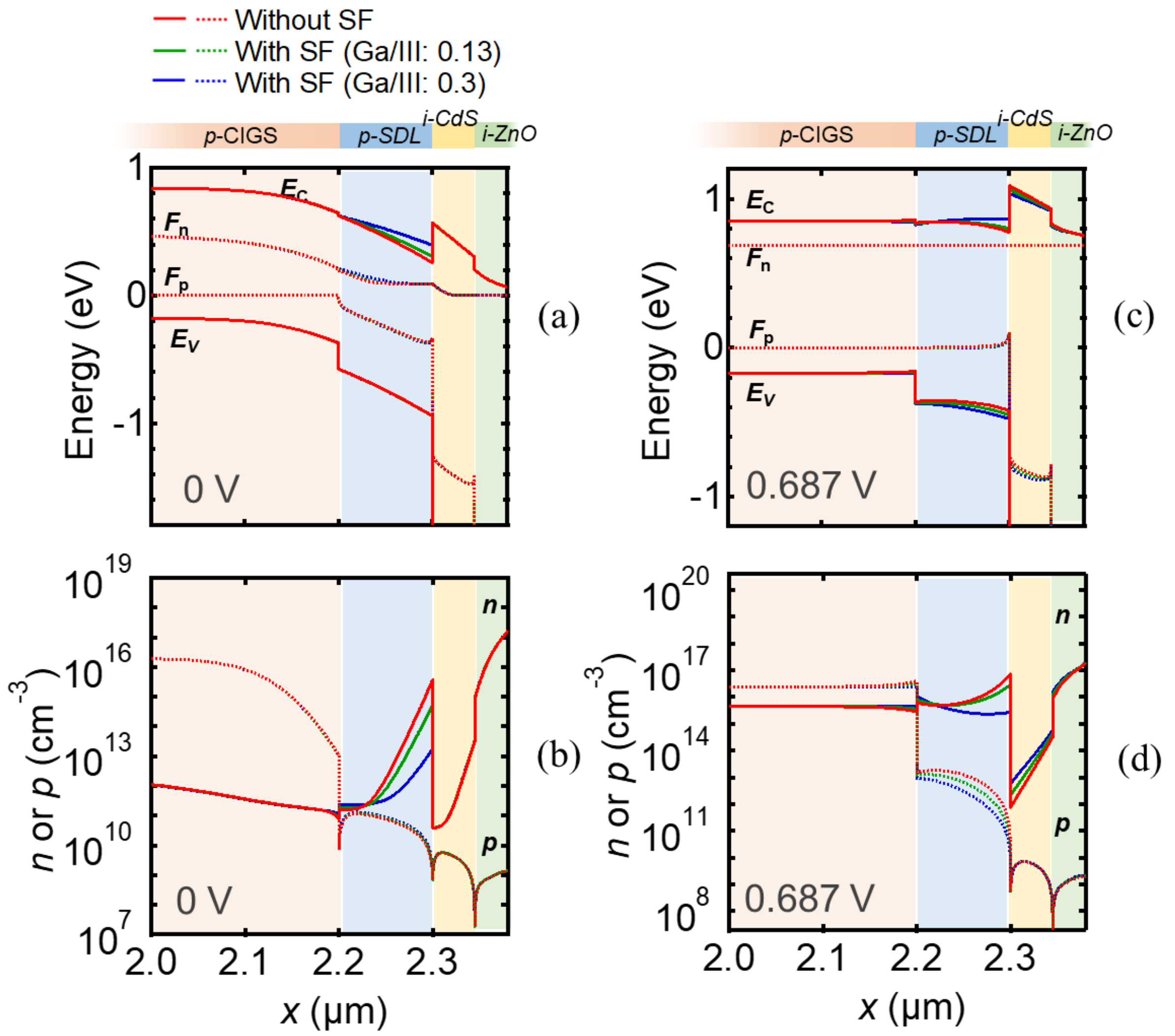

Figure S8. Calculated band diagrams and distribution of electron density (n) and hole density (p) at (a, b) 0 V and (c, d) 0.687 V using SCAPS for CIGS solar cells with and without the SF. In the calculation the conductivity of SDL and CdS were defined as p-type (10^{16} cm^{-3}) and intrinsic, respectively.

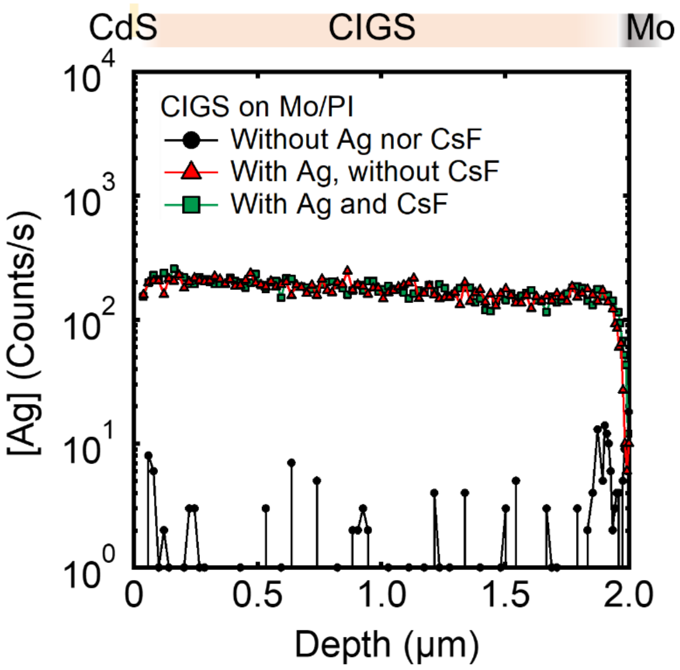

Figure S9. [Ag] in the CIGS layer fabricated without Ag alloying or CsF-PDT, with Ag alloying and without CsF-PDT, with Ag alloying and CsF-PDT on Mo-coated PI substrate evaluated via SIMS.
